# Rethinking flood resilience: systemic risk, governance failures, and the social production of vulnerability in the Himalayan–Indus region

**DOI:** 10.1007/s44367-026-00032-8

**Published:** 2026-04-13

**Authors:** Abdur Rahim Hamidi, James D. Ford, Paula Novo, Jouni Paavola, Ishfaq Hussain Malik

**Affiliations:** 1 Sustainability Research Institute, School of Earth, Environment and Sustainability, University of Leeds, Leeds, United Kingdom; 2 Priestley Centre for Climate Futures, University of Leeds, Leeds, United Kingdom; 3 School of Geography, University of Leeds, Leeds, United Kingdom; 4 Centre for Development and Environment, University of Bern, Bern, Switzerland

**Keywords:** Systemic risk, Social vulnerability, Flood governance, Himalayan–Indus basin, Climate resilience, Disaster politics

## Abstract

Flood disasters in the Himalayan–Indus Basin are increasing in frequency, scale, and impact, driven by glacial melt, erratic monsoons, and changing land-use patterns. Yet their effects are unevenly distributed, and their causes extend beyond climatic or environmental factors. Using the 2022 Pakistan floods as a case study, this paper argues that such disasters are not isolated anomalies but emerge from the intersection of climate extremes, systemic risk, social vulnerability, and governance failure. Drawing on interdisciplinary scholarship in disaster studies, hydropolitics, and critical development, this study employs a qualitative, exploratory research design, analysing published papers, technical and policy reports, and grey literature. A critical social science perspective frames the analysis, highlighting how historical and political processes—including colonial-era infrastructure, socio-political exclusion, and institutional dysfunction—have produced and distributed systemic flood risk in Pakistan. We argue that flood crises stem not only from climate extremes but are systemically produced by historical and political processes, shaped by intersecting hierarchies of social, economic, and political marginalization. The paper critiques dominant hazard-centric and technocratic responses and calls for reimagining resilience as a transformative process—one that involves overhauling governance, dismantling exclusion, rebuilding socio-ecological relations, and embedding equity and justice in disaster risk strategies. The paper advocates for just, participatory, and ecologically grounded approaches to resilience. While focused on Pakistan, the findings offer broader insights for understanding and addressing systemic flood risk across the Himalayan–Indus region and comparable climate-vulnerable contexts. The paper contributes to ongoing debates on disaster governance and resilience in climate-vulnerable regions.

## Introduction

Climate change is reshaping South Asia’s transboundary river systems and amplifying water-related risks. The Himalayan range, holding the largest non-polar ice reserves, feeds South Asia’s densely populated basins, including the Brahmaputra, Indus, and Ganges (Immerzeel et al. 2010, Ahmed 2025), sustaining agriculture, energy generation, and drinking water for millions (Molden et al. 2014, Sharma and Choudhury 2021). However, temperature rise, monsoon dynamics, and extreme events are driving rapid glacier loss, altering hydrology and landscapes with consequences for ecosystems and human societies (Shaw et al. 2022, Almazroui et al. 2020, Douglas 2009). These upstream changes amplify floods in lowland plains, where socio-economic disparities heighten exposure and vulnerability (Mirza 2011), with monsoonal floods often causing cycles of displacement and developmental setbacks (Shaw et al. 2022, Almazroui et al. 2020, Douglas 2009).

Pakistan, located in the lower Indus Basin, exemplifies this multi-scalar vulnerability. Ranking among the top ten countries most affected by climate-linked disasters (Eckstein et al. 2021) and recognized as highly vulnerable and poorly prepared (MPDSI-Pakistan 2022, p.18), it has experienced 28 major floods since independence (1947) (Federal Flood Commission 2021, p.38). The 2022 floods demonstrate how high-mountain climate dynamics and intensified monsoon rainfall interact with infrastructural fragilities and governance shortcomings to produce systemic flood risk. Displacing nearly 8 million people and causing over USD 30 billion in economic losses (MPDSI-Pakistan 2022), the event exposed long‑standing weaknesses shaped by historical development trajectories, uneven state capacities, and socio‑political marginalization (Ali et al. 2019, Masud and Khan 2023). While the empirical focus of this paper is Pakistan, the case is analytically situated within the wider Himalayan–Indus region, where common climatic drivers and historical water‑management legacies shape flood regimes and downstream vulnerabilities across multiple basins. This allows the case to speak to broader debates on transboundary hydrology, climate risk, and disaster governance in comparable mountain‑to‑plains systems.

Beyond the physical hazard, the central question is not how hazards occur, but who suffers most and why. This study goes beyond hazard-centric interpretations to examine Pakistan not merely as a site of climatic extremes but as a context to understand how vulnerability is socially, historically, and politically produced. This analysis is situated within three intersecting literatures that interrogate the socio-political construction of risk. First, disaster studies emphasize that vulnerability is not inherent but socially constructed, rooted in unequal access to resources, political marginalization, and institutional neglect (Wisner et al. 2004, Cutter et al. 2003). Second, hydropolitical scholarship shows how colonial-era infrastructure, centralized water control, and politically mediated distribution systems have shaped long‑term patterns of exposure and vulnerability (Mustafa 2007, Haines 2013, Taylor 2015, Mustafa et al. 2013). Third, critical development perspectives highlight how technocratic, top-down interventions often overlook local realities, reproduce injustices, and reinforce structural risk rather than eliminate it (Wisner et al. 2025, IPCC 2022, Gaillard 2010, Ribot 2014). Together, these perspectives inform the argument that floods in Pakistan—and the broader Himalayan–Indus region—are not simply the outcome of climate hazards or natural anomalies but manifestations of systemic risk produced at the intersection of environmental change, socio‑political hierarchies, and institutional dysfunction.

But it is first important to outline the climatic and hydrological context underpinning Pakistan’s vulnerability. This vulnerability arises from the interaction between a monsoon-dependent climate and glacial hydrology within the Indus River Basin. Seasonal flooding, exacerbated by upstream glacial melt and erratic rainfall (Syvitski and Brakenridge 2013, Ali et al. 2020), has become both frequent and devastating (Webster et al. 2011). Marginalized communities in high-risk floodplains bear the brunt of this exposure due to inadequate housing, food insecurity, and limited adaptive capacity (MPDSI-Pakistan 2022, Government of Pakistan 2022b). While some progress has been made toward proactive disaster risk reduction (DRR) and localized resilience strategies (Khan et al. 2022), Pakistan’s disaster management remains centred on emergency relief and hard infrastructure. Though necessary, they are insufficient in the face of intensifying and recurring climate risks (Rizwan et al. 2023). This context raises critical questions: Why does Pakistan continue to suffer recurrent flood disasters and repeated patterns of damage? What systemic failures—of governance, infrastructure, and social equity—sustain this vulnerability? And what pathways might enable more transformative, equitable flood risk governance?

To explore these questions, we adopt a qualitative, exploratory case study approach and consider the 2022 Pakistan floods as a paradigmatic instance of systemic risk. This approach enables the identification of underlying themes and structural patterns that produce vulnerability, offering insights for developing new critical questions about disaster governance in climate-vulnerable contexts. Through qualitative analysis of secondary sources, including peer-reviewed research, government reports, and international assessments, it investigates how vulnerability is historically produced, politically sustained, and socially differentiated. The documentary evidence is interpreted through a thematic lens to identify recurring narratives around infrastructure, vulnerability, and governance, while inconsistencies across sources are used to highlight institutional blind spots and contested problem framings. This approach enables a deeper interrogation of governance failures, infrastructural legacies, and socio-political exclusions shaping flood exposure and vulnerability. Three central arguments emerge: floods are socially and historically produced through governance failures and institutional legacies; impacts are shaped by intersecting inequalities of gender, geography, and political power; and effective governance must centre ecological resilience, local participation, and decolonial approaches. Although Pakistan is the primary empirical focus, it is analytically situated within the Himalayan–Indus system, where upstream cryospheric change, monsoon dynamics, and hydrological flows shape downstream flood regimes. Pakistan represents a paradigmatic downstream case where regional climatic and hydrological processes intersect with local socio‑political structures, making the case analytically transferable to other Himalayan basin contexts.

In what follows, Section 2 outlines the methodology. Section 3 present the conceptual framework. Section 4 situates Pakistan’s vulnerability within its climatic and hydrological context. Section 5 analyses the 2022 floods as a case of systemic risk. Section 6 examines the climate–water–governance nexus. Section 7 explores the social production and differentiated impacts of flooding. Section 8 brings together theoretical debates in disaster risk, hydro-politics, and critical development studies with insights from Pakistan to outline pathways toward equitable, locally informed, and ecologically grounded flood governance.

## Methodology and approach

Building on the framing established in the introduction, this research adopts an exploratory, qualitative case study design (Swedberg et al. 2020) to investigate the systemic nature of flood risk and vulnerability, using the 2022 Pakistan floods as a paradigmatic case. An exploratory approach is appropriate for unpacking complex, historically rooted governance and vulnerability dynamics that cannot be captured through quantitative indicators alone. The approach emphasizes analytical depth, process‑tracing, and interpretive synthesis over representativeness. The analysis draws on qualitative secondary and documentary data, including academic literature, grey literature, government documents, and reports from international agencies such as the UN and World Bank. The sources were selected to facilitate triangulation and to capture diverse perspectives on disaster risk, policy responses, and socio-political context.

To enhance transparency, sources were selected using three criteria: *relevance* (documents had to substantively address systemic risk, flood governance, vulnerability, climate–water interactions, or institutional arrangements), *credibility and traceability* (priority was given to peer‑reviewed studies, official policy documents, and institutional reports with clearly identifiable evidence bases and methodological grounding), and *temporal alignment* (the focus was primarily on works from the past two decades to ensure relevance to current policy and climate realities, while including older policy documents or historical accounts where necessary to trace the evolution of institutional legacies and infrastructural systems).

The materials were subjected to critical thematic analysis of recurring narratives and patterns surrounding governance, infrastructure, and social marginalization. Rather than simply cataloguing impacts or responses, the analysis interrogated how risk is constructed, distributed, and normalized in dominant discourses. All selected materials were examined through qualitative thematic analysis to identify cross‑cutting patterns and divergences. *Initial reading and coding* of documents to identify recurring narrative strands related to infrastructural performance, governance coordination, vulnerability production, and policy framings. *Iterative clustering of codes* into broader themes—such as systemic risk drivers, social differentiation, hydropolitical legacies, and institutional fragmentation—guided by the conceptual framework. An *intersectional lens* examined how vulnerability differs across gender, class, caste, and geographic lines. Special attention was given to policy and governance discourses—such as resilience, disaster management, relief, and climate adaptation. Triangulation across academic, governmental, and international assessments to strengthen credibility and reduce reliance on any single narrative or institutional perspective. The approach is interpretive rather than enumerative: it does not quantify impacts but instead synthesizes structural, institutional, and discursive patterns across documentary evidence.

Given the reliance on documentary sources, the analysis does not claim to represent lived experiences directly nor to substitute for ethnographic or household‑level data. Instead, it reconstructs the structural and institutional context shaping vulnerability and governance trajectories. This approach is appropriate for examining long‑term infrastructural legacies, policy evolution, and systemic governance failures. While the study is primarily focused on Pakistan, documents concerning regional monsoon dynamics, Himalayan cryosphere change, and transboundary water governance are incorporated where they illuminate basin‑scale processes relevant to systemic risk in the lower Indus Basin. The goal is not to generalize across countries but to situate the case within a broader Himalayan–Indus hydrological and governance context.

## Conceptual framework

**Fig. 1:**
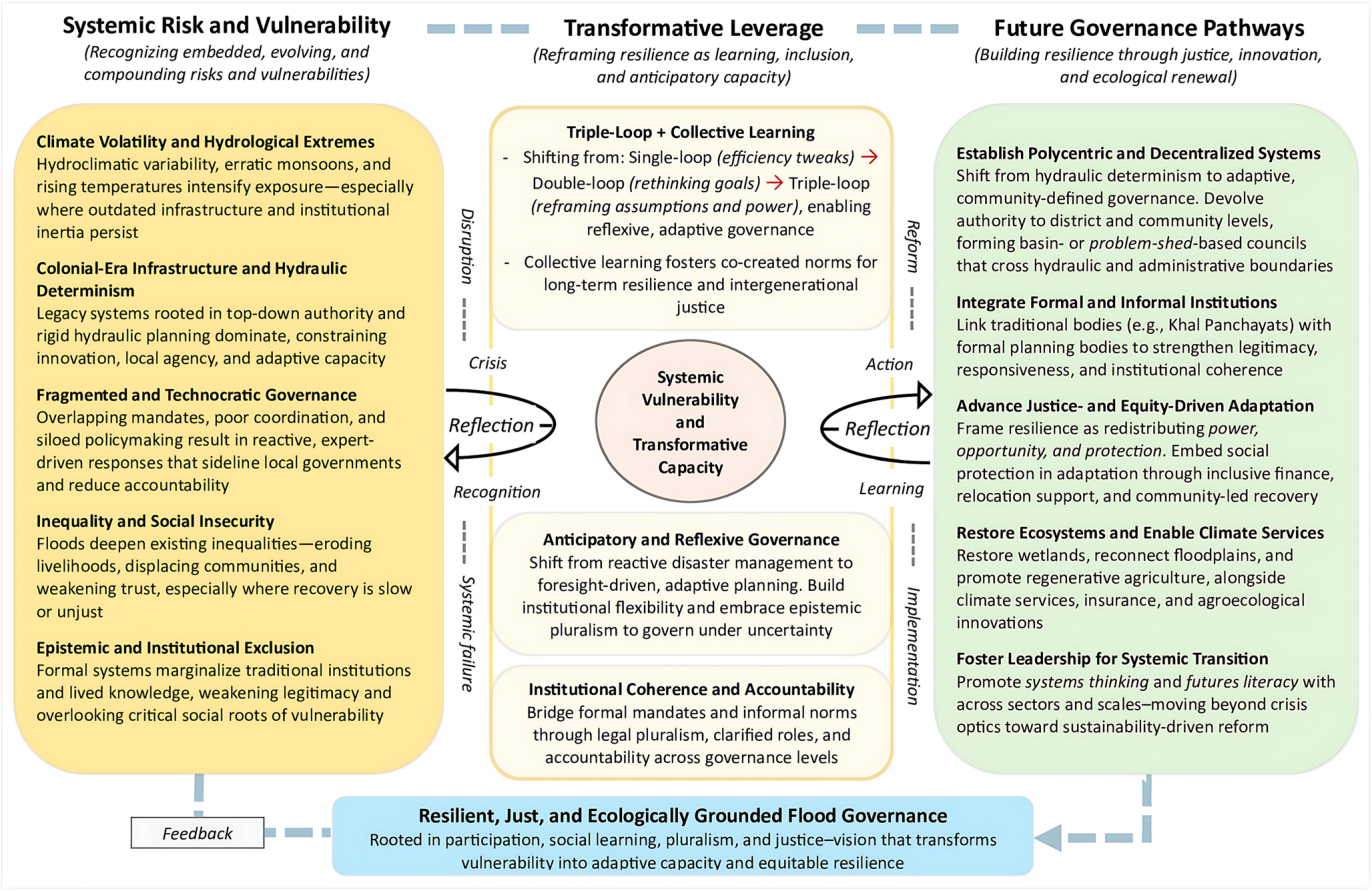
Conceptual framework for transformative flood governance. The framework is structured around three interlinked components—systemic risks, transformative leverage, and adaptive governance pathways—and emphasizes justice, decentralization, institutional coherence, and inclusivity as foundations for building equitable resilience

This paper draws on a conceptual framework developed to understand how flood risk is socially produced and how it might be transformed. The framework (Fig. 1) outlines a future-oriented model for transformative flood governance, synthesizing systemic drivers of vulnerability, identifying leverage points for institutional learning and change, and proposing actionable governance pathways. Four concepts are analytically distinguished. *Systemic risk* refers to risks that emerge from interdependent and cascading interactions between climatic extremes, infrastructural weaknesses, and institutional fragmentation. Such risks transcend individual sectors and generate failures across governance, infrastructure, livelihoods, and social systems. In this sense, systemic risk is not the sum of isolated shocks but the product of their interactions—particularly evident when glacial melt, monsoon variability, and poorly maintained hydraulic infrastructure converge to amplify downstream exposure. *Social vulnerability* captures the historically produced socio‑economic and political conditions that shape differential exposure, sensitivity, and recovery capacity. Vulnerability is rooted in inequalities of land access, wealth, gender, caste, and political power, and is reproduced through uneven development and exclusionary governance. It therefore emphasizes who is at risk and why—focusing attention on the structural factors that determine which groups bear the brunt of flood impacts. This understanding reflects longstanding work in disaster studies that views vulnerability as socially constructed rather than inherent (Wisner et al. 2025, Tierney 2025, Kelman 2020).

*Governance failure* denotes institutional misalignment, fragmentation, limited coordination, and entrenched power relations that restrict the effective management of risk. Governance failure is not simply the absence of capacity; it also includes the political incentives and institutional histories that produce uneven protection and reactive, hazard‑centric responses. Such failures are seen in overlapping mandates, weak early warning dissemination, poor maintenance of infrastructure, and a persistent emphasis on structural interventions at the expense of local participation. *Transformative resilience* refers to long‑term, structural reconfigurations of governance systems that address underlying inequities and social vulnerabilities instead of merely restoring pre‑disaster conditions. Transformative resilience emphasizes anticipatory governance, institutional learning, and justice‑centred approaches. It moves beyond incremental improvements by targeting power relations, institutional fragmentation, and ecological degradation as central drivers of flood risk. These four concepts are interrelated yet analytically distinct. Systemic risk explains the multi-scalar dynamics through which climate processes and institutional fragilities combine; social vulnerability highlights inequalities shaping differential impacts; governance failure identifies institutional roots of persistent exposure; and transformative resilience offers a future‑oriented vision for equitable reform.

At its core, the framework treats flood risk as historically, socially, and politically constructed rather than purely natural. It identifies drivers of systemic risk—including fragmented governance, entrenched inequalities, political exclusion, erosion of local institutions, and aging hydraulic infrastructure—that interact to amplify impacts and concentrate risk among marginalized populations. Within this model, transformative leverage points are highlighted, emphasizing the need for participatory governance, envisioned through integration of formal and informal institutions (Mollinga et al. 2007, Mustafa and Wrathall 2011, Munene et al. 2018), justice-centred resilience (Hofbauer et al. 2025, Hamdanieh et al. 2024), ecosystem- and nature-based adaptation (Turner et al. 2022, McVittie et al. 2018, Muthee et al. 2021), and multi-scalar coordination (Gioia and Gioia 2024). These leverage points draw on triple-loop learning (Pahl-Wostl 2009) and anticipatory governance to shift from reactive, emergency-focused responses toward adaptive, forward-looking strategies (Umbach 2024). Finally, the framework outlines adaptive governance pathways centred on decentralization, institutional coherence, inclusivity, and context‑specific governance approaches. Concepts like ‘problem-sheds’ (Mollinga et al. 2007, Woodhouse and Muller 2017)—an alternative to watersheds (Fisher 1967, Thomas 2020)—promote governance aligned with locally relevant water challenges rather than rigid administrative or watershed boundaries, enabling context-specific and cross-sectoral water governance (Giordano and Shah 2014, Mollinga 2020). These pathways connect local knowledge, ecosystem‑based adaptation, and polycentric coordination to reimagine flood governance as a collaborative, equity‑driven process.

The conceptual framework serves two analytical functions: *interpretive lens* guides the reading of documentary sources by identifying how infrastructures, institutions, and socio-political structures co-produce vulnerability and risk; and *diagnostic and forward-looking lens* provides a basis for evaluating the 2022 floods not simply as environmental events but as outcomes of long-term governance failures, supporting the development of equitable, ecologically grounded policy recommendations.

## The Himalayan–Indus region: climate and hydrological context

This section situates Pakistan as a paradigmatic downstream case in a larger Himalayan–Indus system, clarifying how basin‑scale climatic and cryospheric processes interact with downstream hydrology and exposure. Stretching over 2,400 km across South Asia, the Himalayan range represents one of the world’s most diverse and complex mountain systems in terms of climate and hydrology. Shared by India, Nepal, Bhutan, China (Tibet), and Pakistan, the region covers about 595,000 km² and exhibits sharp contrasts in climate and glacier dynamics. Climatic influences show pronounced variation across the region—the Eastern Himalaya is receiving the highest precipitation dominated by intense monsoonal rainfall, the Central Himalaya reflects a combination of summer monsoon and winter westerly influences, while the Western Himalaya remains largely arid to semi-arid under westerly influence. These contrasting regimes produce distinct glacier accumulation and melt patterns, shaping diverse hydrological responses across the region (Ahmed 2025, Scherler et al. 2011). Hydrologically, the Himalaya feeds the Brahmaputra, Ganges, and Indus rivers, supplying water to almost 1.5 billion people across South Asia (Shrestha et al. 2015). The Indus Basin draws mainly from glacier and snowmelt, while the Ganga and Brahmaputra depend more on monsoonal precipitation, with smaller cryospheric contributions (Nepal and Shrestha 2015). Often called the “Third Pole,” the Himalaya holds the largest non-polar ice reserves (Gao et al. 2024, Zheng et al. 2021), serving as a crucial freshwater reserve (Azam et al. 2021, Molden et al. 2022), regulating flows and supporting ecosystems, agriculture, and livelihoods (Azam et al. 2021, Nie et al. 2021, Kulkarni et al. 2021, Negi et al. 2017).

Glacial retreat has accelerated across the region, varying with local climate and topography (Kulkarni et al. 2021, Clason et al. 2022, Ji et al. 2022). Glaciers in the eastern and central Himalayas, primarily fed by monsoonal rains, are retreating faster compared with the western Himalayas, where snow falls in the winter months from mid-latitude westerlies maintains more stable mass balances (Ahmed 2025, Fugger et al. 2022). Observed 21 st century warming ranges between 0.15 °C and 0.60 °C per ten-year period—surpassing the global average (Shrestha et al. 2015, Negi et al. 2017). Melt rates have accelerated: Gangotri glacier retreating 16–18 m/year on average (Singh et al. 2022, Aiyar and Raina 2022), Masar glacier 49 m/year, and Khatling and Phating glaciers 29.5 m/year and 16.6 m/year, respectively (Banerjee et al. 2024). On average, Himalayan glaciers retreat 14.9 ± 15.1 m/year, with basin-level variations of 12.7 ± 13.2 m/year (Indus), 15.5 ± 14.4 m/year (Ganga), and 20.2 ± 19.7 m/year (Brahmaputra) (Ahmed 2025).

Projections indicate temperature increases of 2 °C to 5 °C by 2100, depending on emission scenarios (Rangwala et al. 2020, Dimri et al. 2021). High-emission pathways (RCP 8.5) could reduce Himalayan glacier volume by over 60% (Zhang et al. 2016), while moderate scenarios (RCP 4.5) predict losses up to 35% (Nie et al. 2021). During glaciers retreat, a phase of increased meltwater—“peak water”—may temporarily boost downstream flows, followed by long-term decline and greater seasonal variability (Ahmed 2025, Kundzewicz et al. 2019). These dynamics are particularly consequential for the Indus Basin, given its strong cryospheric dependence. Because snow and glacier melt contribute a large share of Indus discharge in Pakistan, predominantly in the summer season, the basin is highly sensitive to shifts in both cryospheric and monsoon processes (Biemans et al. 2019, Abbasi 2017). These dynamics will intensify both flooding during melt seasons and water scarcity in dry periods, trends that heighten intra‑annual variability and complicate water management. For Pakistan, this means not only heightened flood hazard during melt and monsoon periods but also greater volatility in dry‑season water availability—conditions that interact with social vulnerability and governance constraints discussed in subsequent sections.

The regional context presented here does not claim basin‑wide uniformity; rather, it clarifies why Pakistan’s downstream exposure is shaped by upstream cryosphere–monsoon dynamics and why floods such as in 2022 arise from interactions across physical, social, and institutional systems. This sets up our case analysis of the 2022 event as an instance of systemic risk: cross‑scalar climatic drivers coupled with infrastructure limits and fragmented governance.

## Flooding as a systemic risk: Pakistan’s 2022 disaster in focus

Over the past seven decades, Pakistan has witnessed recurring devastating flood events. Table 1 shows major floods between 1950 and 2022, highlighting their toll of lives lost, villages affected, flooded area, and direct economic losses. The data illustrates not only the frequency of such disasters but also their escalating intensity and scale. The 2010 and 2022 floods alone accounted for over US$ 40 billion in losses. This trajectory shows that flooding is a long-standing crisis that continues to evolve under a changing climate.

**Table 1:** Major flood events witnessed in Pakistan (1950–2022)

S.No.	Year	Direct Losses (US$ Million)	Lives Lost	Affected Villages	Flooded Area (Sq. km)
1	1950	488	2,190	10,000	17,920
2	1955	378	679	6,945	20,480
3	1956	318	160	11,609	74,406
4	1957	301	83	4,498	16,003
5	1959	234	88	3,902	10,424
6	1973	5,134	474	9,719	41,472
7	1975	684	126	8,628	34,931
8	1976	3,485	425	18,390	81,920
9	1977	338	848	2,185	4,657
10	1978	2,227	393	9,199	30,597
11	1981	299	82	2,071	4,191
12	1983	135	39	643	1,882
13	1984	75	42	251	1,093
14	1988	858	508	100	6,144
15	1992	3,010	1,008	13,208	38,758
16	1994	843	431	1,622	5,568
17	1995	376	591	6,852	16,686
18	2010	10,056 @1US$ = PKR 86	1,985	17,553	160,000
19	2011	3,730 @1US$ = PKR 94	516	38,700	27,581
20	2012	2,640 @1US$ = PKR 95	571	14,159	4,746
21	2013	2,000 @1US$ = PKR 98	333	8,297	4,483
22	2014	440 @1US$ = PKR 101	367	4,065	9,779
23	2015	170 @1US$ = PKR 105	238	4,634	2,877
24	2016	6 @1US$ = PKR 104.81	153	43	–
25	2017	–	172	–	–
26	2018	–	88	–	–
27	2019	–	235	–	–
28	2020	–	409	–	–
29	2021	–	198	–	–
30	2022	30,000* @1US$= PKR 225	1,739	6,631^	85,000`
31	2023	–	226	–	–
32	2024	–	368	–	–
Total		**68**,**225**	**23**,**982**	**203**,**704**	**701**,**558**

Note: ^ Union Councils (administrative units), `United Nations Satellite Centre: Floods Imagery Analysis from 1.7.2022 to 31.9. 2022. Source: Federal Flood Commission Annual Report-2024 (Federal Flood Commission 2024), National Disaster Management Authority (NDMA 2024), *Post-Disaster Needs Assessment Report-2022 (Government of Pakistan 2022a)

**Fig. 2:**
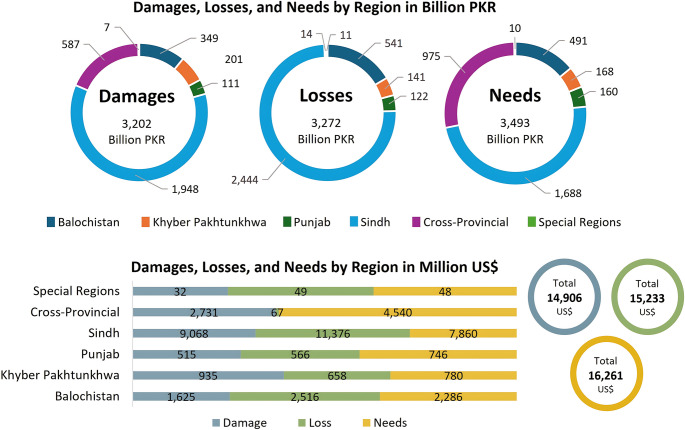
Damage, loss, and needs by region

The 2022 floods inundating nearly one-third of the country, affecting 33 million people, and displacing nearly 8 million (MPDSI-Pakistan 2022), were a tipping point. They exposed vulnerabilities shaped by governance failures, infrastructural neglect, and deep-rooted inequality. This section reframes the event as a case of systemic risk: extreme rainfall, glacial melt, and La Niña-linked atmospheric anomalies triggered cascading socio-economic and institutional failures. Yet the devastation was neither unexpected nor purely environmental—it resulted from climatic shocks intersecting with weak infrastructure, fragmented institutions, and social inequalities. The economic toll exceeded $30 billion in total, with reconstruction needs over $16 billion (Fig. 2). Sindh was the hardest hit, followed by Balochistan and Khyber Pakhtunkhwa (MPDSI-Pakistan 2022). These figures underscore both the scale of devastation and the immense challenge of recovery.

**Fig. 3:**
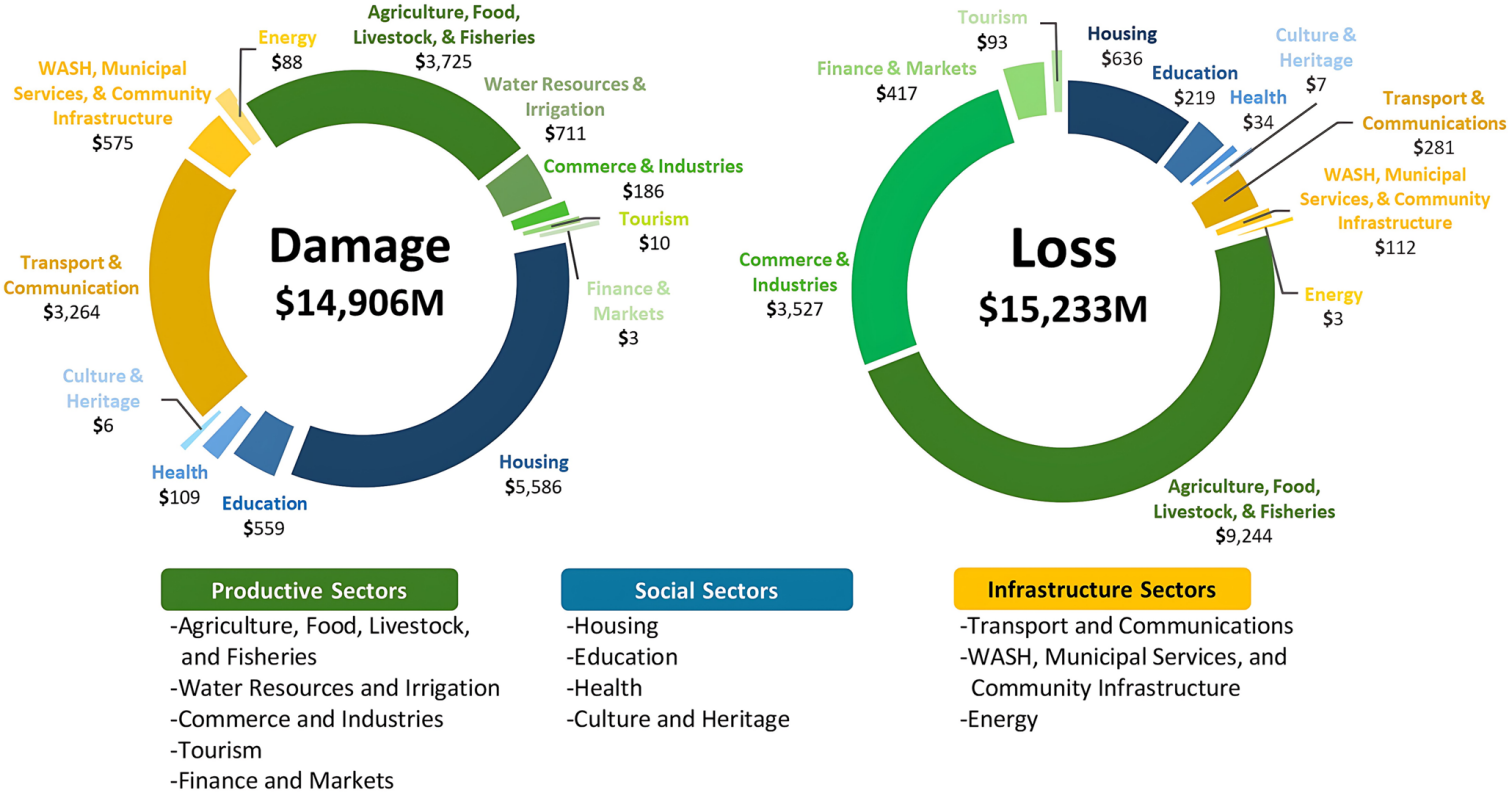
Distribution of damage and loss by sector

Agriculture, nearly a quarter of GDP (PBS 2023) and employs over a third of the workforce (PBS 2021), suffered $3,725 million in damages and $9,244 million in losses (MPDSI-Pakistan 2022; NDMA 2024), as shown in Fig. 3. Over 6.5 million acres of crops were destroyed; Sindh alone lost nearly 90% of cotton crop and 61% of sugarcane (Government of Pakistan 2022b, p.20, Qamer et al. 2023). Agriculture bore $12.9 billion in combined damages and losses, disproportionately affecting smallholders and rural labourers living in precarious conditions (MPDSI-Pakistan 2022), with many struggling to meet their food needs (IRC 2022). Housing suffered over $5.5 billion in damages, while education and health incurred millions more (MPDSI-Pakistan 2022; NDMA 2024).

Before the floods, 21.8 million people were employed in affected districts, 43% engaged in agriculture (Table 2). Over 4.3 million people lost livelihoods, mostly in agriculture (PBS 2022, MPDSI-Pakistan 2022, p.143), with three-quarters of households unable to find work post-flood (Government of Pakistan 2022b, p.20), revealing the compounding social impacts of the disaster.

**Table 2:** Impact on livelihoods: job losses and income reduction

	Number of People
Pre-flood employment	21.8 million
Employment in agriculture	9.4 million (43% of pre-flood workforce)
Affected by job losses/disruptions	4.3 million (20% of pre-flood workforce)
Affected workers with livestock/agricultural land	3.7 million

Author’s own table. Source: MPDSI-Pakistan (2022, p.143)

The destruction of livelihoods intensified pre-existing food insecurity. Over 7 million households in were food insecure before floods (MPDSI-Pakistan 2022, p.23; PBS 2021). Post-flood estimates rose to 14.6 million (Table 3) (MPDSI-Pakistan 2022, p.24). Crop, livestock, and food-stock losses, combined with inflation and disrupted supply chains, undermined food access and affordability. In the first half of 2023, food inflation soared to 45%, limiting the ability of low-income households to access food (UNOCHA 2023).

**Table 3:** Household’s flood insecurity before and after 2022 floods

Province	Food Insecure Households (pre-flood)	Estimated Food Insecure Households (post-flood)	Total Estimated Food Insecure Households
Sindh	3.9 million	4.3 million	8.2 million
Balochistan	1.6 million	0.8 million	2.4 million
KP	0.6 million	1.7 million	2.3 million
Punjab	0.8 million	0.9 million	1.7 million
Total	**7 million**	**7.6 million**	**14.6 million**

Author’s own table. Source: MPDSI-Pakistan (2022, p.23–24)

Over 2 million homes were damaged or destroyed, displacing millions (Harneis 2023). Emergency shelters were overwhelmed or non-existent, forcing many to makeshift roadside encampments or under open skies for weeks. About 13% of healthcare facilities were destroyed, compromising immunization, maternal care, and emergency services. Access to clean water, sanitation, hygiene, and healthcare was severely limited (MPDSI-Pakistan 2022, Moazzam 2023). Women and girls faced heightened risks, including lack of maternal healthcare, gender-based violence, and unsafe conditions in overcrowded emergency shelters (Tufail et al. 2023; UNFPA 2022). The destruction of 27,000 schools affected 2 million children, increasing child labour, early marriage, and intergenerational poverty. For many girls, the floods marked a permanent interruption in education (Dahlin and Barón 2023; UNICEF 2022) and increased the likelihood of child marriage—reported to be rising in some districts as families turned to harmful coping strategies (Seigneur 2024).

**Fig. 4:**
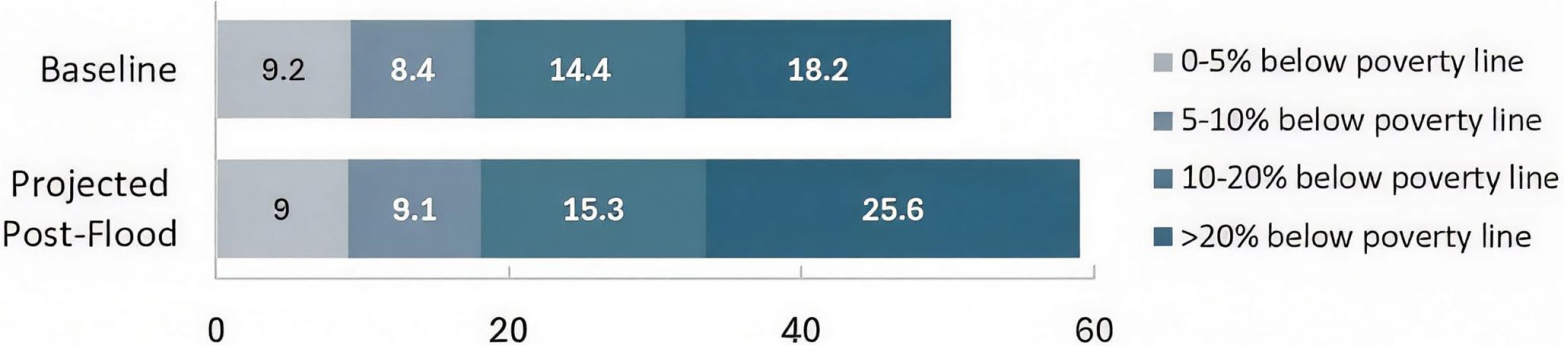
Number of people living more than 20% below the poverty line (millions)

Poverty worsened: floods hit the poorest hardest (Rana et al. 2023), as poverty and vulnerability are closely linked (Hamidi et al. 2020, Sinha et al. 2022). While poverty can increase vulnerability to the impacts of floods, it can also be further worsened by flood impacts (Dube et al. 2018). Pre-flood, most affected districts already were more deprived: 19 of the 25 poorest districts were impacted, with poverty rates 10 points above the national average (31.4% vs. 21.9%) (MPDSI-Pakistan 2022, p.143). Approximately 8.73 million people fell below the poverty line, with Sindh hardest hit (4.88 million). Extremely poor populations, living more than 20% below poverty line, projected to increase from 18 to 25.6 million (MPDSI-Pakistan 2022, p.20) (Fig. 4).

Rural and peri-urban districts with weak infrastructure and highest poverty rates suffered the greatest losses. In these areas, floodwaters did not just wash away homes and crops—they exposed the fragile social contract and systemic vulnerabilities. Employment in agriculture and construction saw the highest simulated poverty increases, underscoring how flood exposure compounds existing economic precarity.

The 2022 floods confirm that vulnerability, not hazards alone, drives disaster. Weak coordination, reactive management, and underfunded social protection systems left communities exposed (Harvey et al. 2022), while local governments struggled with early warnings, relief, and recovery. What began as a climatic event became a multidimensional governance crisis (Zafar 2022). Climate shocks intersected with socio-political fault lines, producing cascading failures across scales and sectors. Flooding in Pakistan is thus a structural phenomenon shaped by inequitable governance and exclusion. Understanding the 2022 disaster requires examining the climatic, hydrological, and institutional drivers—monsoon variability, glacial dynamics, water governance, infrastructural legacies, and unequal vulnerability that determine its severity and reach.

## The climate–water–governance nexus

Floods in Pakistan are no longer rare events—they are frequent, intensifying, and symptomatic of deeper systemic fragilities. Climate change is accelerating glacial melt, disrupting monsoon patterns, and amplifying extremes, while outdated infrastructure and fragmented governance leave millions exposed. The 2022 floods were not an isolated catastrophe but the product of intertwined environmental, social, and institutional weaknesses.

### Climate change impacts and future projections

**Fig. 5:**
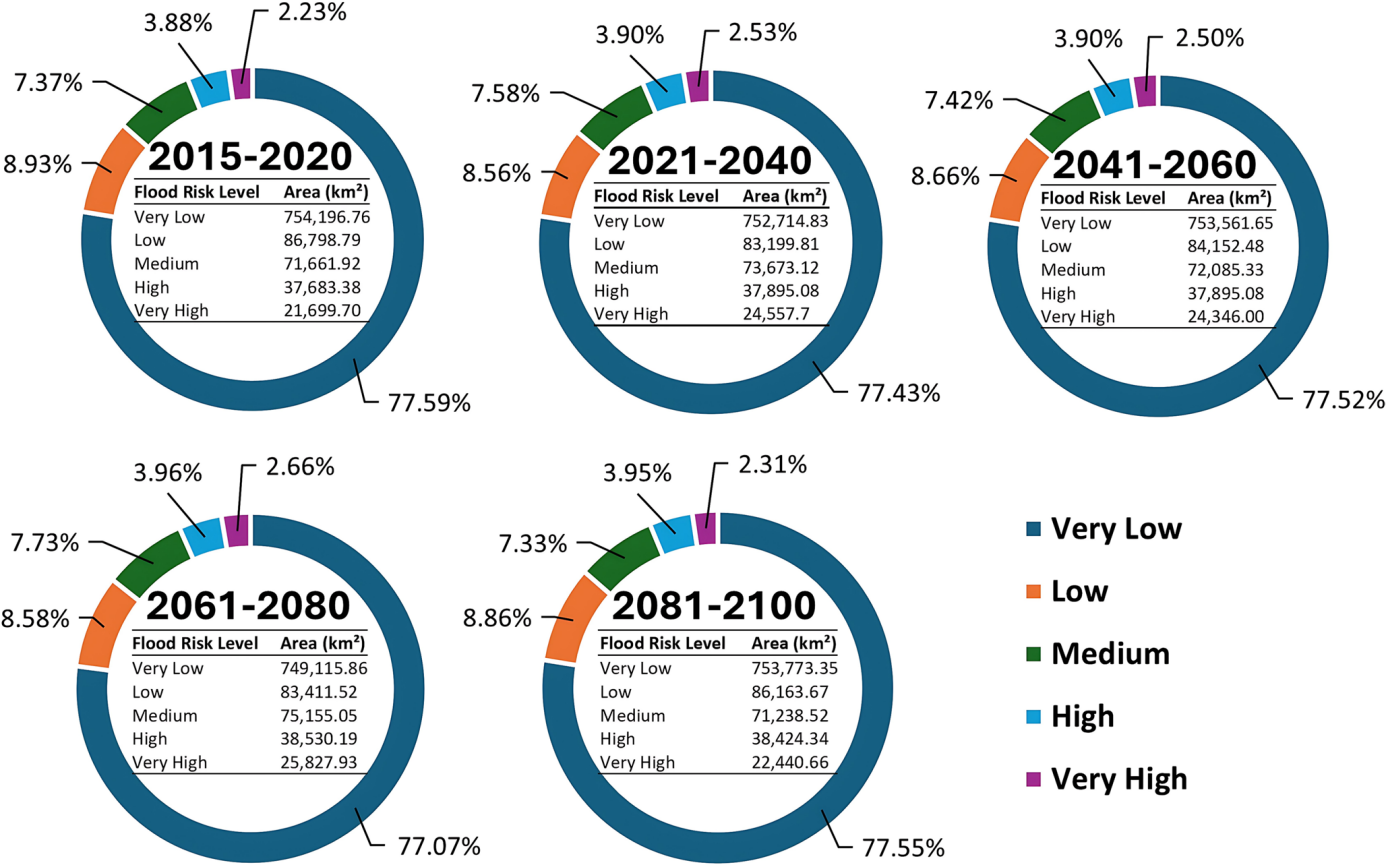
Area (km^2^) and percentage (%) of flood risk area of 2015–2100 in Pakistan

Pakistan’s flood risk is inextricably linked to shifts in its climate and hydrology—particularly within the glacier-fed Indus Basin. Under high-emission scenarios, up to 80% of glacier volume could vanish by 2100 (Wester et al. 2019, ICIMOD). Accelerated warming across the Tibetan Plateau (Qin et al. 2009, Rangwala et al. 2009) and elevation-dependent processes (Rangwala et al. 2009, Palazzi et al. 2017) are already disrupting the volume, timing, and reliability of river flows (Immerzeel et al. 2010, Yang et al. 2014, Wang et al. 2015). Snow and glacier melt contribute 60–70% of Indus River discharge (Biemans et al. 2019), mainly (about 80%) in summer (Abbasi 2017), making flows increasingly climate-sensitive. Regional models project more frequent, intense rainfall in the Upper Indus Basin (Amin et al. 2017, Abbas et al. 2022, Ali et al. 2021), and drying in the Lower Indus (Rajbhandari et al. 2015), exacerbating water imbalances. These shifts are already unfolding: between 2000 and 2015, flood exposure increased by 50% (Tellman et al. 2021); by 2030, over 5.7 million people may face annual flood risk (Burke 2023), rising by another 5 million between 2035 and 2044 (Akram et al. 2024). Flood risk area data for the period 2015 to 2100 (Fig. 5) has a gradual upward trend in the higher risk categories. The “Very High” flood-risk zone is projected to expand from 21,699.70 km² in 2015–2020 to 25,827.93 km² in 2061–2080 (Cui et al. 2025). Approximately 29% of Pakistan’s area—home to 95 million people—is now critically flood-prone, with population exposure estimates 30% higher than previously reported (Waleed and Sajjad 2025). These trends underscore the mounting threat under evolving climatic conditions.

The 2022 floods vividly illustrate these evolving risks. Extreme spring heat triggered premature snowmelt (Mallapaty 2022), saturating rivers before a hyperactive monsoon, amplified by La Niña, ocean warming, and Bay of Bengal depressions (Mallapaty 2022, Hong et al. 2023, Qureshi et al. 2023). The Pakistan Meteorological Department predicted 35–40% above-average rainfall, actual rainfall exceeded 175% of normal. Table 4 presents rainfall data by province, highlighting the intensity of rainfall. August rainfall in Sindh and Balochistan exceeded historical averages by 726% and 590%, respectively (Pakistan Meteorological Department 2022). Four major monsoon spells (June 29–July 9, July 23–28, August 5–13, and August 23–26) and early pre-monsoon rains (June 13–25) overwhelmed river and aging infrastructure, breaching embankments and inundating vast areas. For instance, in August 23,762 km² (88.3%) experienced shallow to moderate flooding (0–2 m), 1,399 km² (5.2%) deep flooding (2–5 m), and 1,752 km² (6.5%) severe flooding (> 5 m) (Cui et al. 2025).

**Table 4:** Rainfall experienced Province-wise – Floods 2022

S.No.	Province	Rainfall Occurred (mm)	% Above Normal
1	Sindh	703.1	426%
2	Balochistan	320.7	450%
3	Punjab	393.5	70%
4	Khyber Pakhtunkhwa	341.1	33%
5	Gilgit-Baltistan	81.1	104%
6	Azad Jammu & Kashmir	382.6	2%

Source: Federal Flood Commission, 2022

The implications extend beyond hydrology, threatening economic stability and livelihoods. Agriculture, dependent on the Indus irrigation system for nearly 80% of its output—making it acutely sensitive to shifts in river flows and precipitation patterns—was hardest hit. The 2022 floods devastated croplands, displaced communities, and disrupted harvests, pushing smallholders into debt and informal off-farm work. Looking ahead, rising temperatures further threaten agricultural labour: a 2 °C rise could reduce labour productivity by over 7% by 2050 (Abeysekara et al. 2024, Roson and Sartori 2016). These climatic pressures intersect with governance: centralized, technocratic, and reactive water management has failed to adapt, leaving risk unmitigated. Water-related challenges are governance-driven (Ringler and Anwar 2013, Young 2019), and the 2022 floods made clear the widening gap between intensifying climate risks and inadequate state response (Shah et al. 2019, Majeed 2023, Aslam 2018), a theme explored further in the section “institutional fragmentation and systemic governance failures”.

In sum, future flooding is certain (Rizwan et al. 2023, Ali et al. 2021, Mehmood et al. 2021). The problem lies not in the hazards themselves, but in the failure to anticipate, adapt, and protect. Scientific evidence is clear: warming is accelerating, glaciers are retreating, and precipitation is becoming erratic. Yet the danger is how these changes interact with rigid infrastructure, inequality, and institutional weakness, transforming climate extremes into humanitarian crises.

### Infrastructures of risk: water management challenges

Pakistan’s flood vulnerability cannot be understood without examining the historical design, degradation, and governance of its water infrastructure. Colonial-era, top-down systems—built to maximize agricultural revenue and control—dominate flood management, remaining poorly adapted to contemporary climate volatility (Mustafa 2007, Haines 2013, Taylor 2015). The hydrological network centres on three main basins: the Makran Coastal, Kharan, and Indus River Basin—the latter covering nearly 40% of the country and sustaining over 80% of irrigated agriculture (Tariq and Giesen 2012; WBG and ADB 2021). Fed by the Jhelum, Chenab, Ravi, Sutlej, and Kabul Rivers, the Indus is managed through a vast network of canals and barrages (Qureshi 2011, Iqbal et al. 2018). Complementing this is 140 reservoirs (e.g., Tarbela, Mangla, Chashma) that stores about 18.92 million acre-feet of water (Tehsin et al. 2019) and divert over 60% of surface flows for agricultural, domestic, and industrial use (Young 2019). Yet during mega-floods like 2010 and 2022, the system proved incapable of managing extreme discharge. Peak flows at major river control points (Table 5) illustrate the scale of hydrological stress: several river stations—particularly along the Indus and Chenab—experienced high to very high flood levels.

**Table 5:** Historic peak discharges (Cusecs) observed in major rivers during 2010 and 2022 flood years

River	Control Point	Design Capacity	2010 Peak Discharge (Cusecs)	2022 Peak Discharge (Cusecs)	2022 Flood Classification
Indus	Tarbela	1,500,000	604,000 (30-7-2010)	418,600 (26-8-2022)	Medium
	Kalabagh	950,000	936,453 (30-7-2010)	423,000 (28-8-2022)	Medium
	Chashma	950,000	1,036,673 (2–8-2010)	523,937 (28-8-2022)	High Flood
	Taunsa	1,100,000	959,991 (2–8-2010)	622,000 (30-8-2022)	High Flood
	Guddu	1,200,000	1,148,200 (8-8-2010)	576,000 (23-8-2022)	High Flood
	Sukkur	900,000	1,108,795 (10-8-2010)	580,000 (25-8-2022)	High Flood
	Kotri	850,000	939,442 (27-8-2010)	600,000 (10-9-2022)	High Flood
Kabul	Nowshera	–	–	336,461 (28-8-2022)	Very High
	Warsak	–	–	–	High Flood
Jhelum	Mangla	1,060,000	2,49,100 (10-8-2010)	139,086 (27-8-2022)	Normal
	Rasul	850,000	2,25,496 (30-7-2010)	40,720 (1–7-2022)	Normal
Chenab	Marala	1,100,000	2,63,795 (30-7-2010)	23,610 (22-6-2022)	High Flood
	Khanki	800,000	2,82,418 (6–8-2010)	210,936 (28-7-2022)	High Flood
	Qadirabad	900,000	3,27,637 (7–8-2010)	210,945 (12-8-2022)	High Flood
	Trimmu	645,000	3,19,733 (7–8-2010)	202,000 (12-8-2022)	Normal
	Panjnad	700,000	3,23,026 (11-8-2010)	112,891 (14-8-2022)	Normal
Ravi	Madhopur	–	3,10,117 (13-8-2010)	–	–
	Jassar	275,000	21,100 (21-8-2010)	112,564 (3-8-2022)	–
	Ravi Syphon	450,000	41,200 (21-8-210)	63,720 (16-8-2022)	–
	Shahdara	250,000	41,900 (21-8-2010)	31,415 (2–8-2022)	–
	Balloki	225,000	41,200 (23-8-2010)	35,235 (3–8-2022)	Normal
	Sidhani	150,000	16,800 (28-7-2010)	21,642 (27-7-2022)	Normal
Sutlej	Sulemanki	325,000	44,300 (30-9-2010)	17,462 (19-7-2022)	Normal
	Islam	300,000	28,900 (20-9-2010)	12,501 (23-7-2022)	–

Source: Federal Flood Commission (Federal Flood Commission 2024), National Disaster Management Authority (NDMA 2024)

These flood peaks underscored not just climatic intensity but also chronic under-preparedness. Pakistan has prioritized water conservation through small dams and check structures to support irrigation. In 2022, 177 dams were damaged, 69 completely breached, mainly built on unsustainable sites, accounted for 80–85% of total economic losses: causing 75% of agricultural damage, compared to 25% from direct rainfall. Seepage, erosion, and overtopping triggered downstream surges, that destroyed 590,440 acres of cropland, 346,303 homes, and 292,526 livestock, amounting to PKR 349 billion (US$ 1.63 billion) in losses. Floodwaters merged with already swollen rivers like the Zhob, Mula, and Hingol—triggering extensive inundation. Out of the 38 calamity-hit districts, 35 were severely affected by dam breaches. In Sindh’s Larkana floodplain, drainage failures formed a 100 km² flash-flood lake, the largest in Pakistan’s history. The southern and central Indus recorded 16,000–18,000 m³/s discharge rates (Cui et al. 2025). Heavy sedimentation from upper-basin gradients has historically raised riverbeds (Manzoor et al. 2013), and the trumpet-shaped lower basin traps floodwaters, prolonging inundation (Cui et al. 2025).

Runoff repeatedly exceeds design capacity, confirming the systems inability to handle mega-floods. Sedimentation, wetland degradation, and poor maintenance have reduced capacity even for moderate flows, with drainage failures now occurring outside flood seasons (Basharat and Rizvi, Mustafa and Wrathall 2011). Structural interventions often redistribute rather than reduce risk—protecting politically influential areas while exposing already vulnerable communities (Taylor 2015, Mustafa et al. 2013, Brohi 2003, Kreutzmann 2011). In the lower Indus Delta, mega-projects like the Right Bank Outfall Drain (RBOD) and Left Bank Outfall Drain (LBOD), built to divert floodwaters, have repeatedly failed (Mahessar et al. 2019). During the 2010 floods, for instance, LBOD backflows worsened flooding in Sindh’s Badin District (Siddiqi 2019). Such outcomes reflect a governance model prioritizing large-scale engineering solutions over adaptive management (Aijaz and Akhter 2020, Akhter 2015, Akhter 2022). Thus, two central drivers underpin current risk and vulnerability: a state-centric approach and continued reliance on outdated systems, both shaped by political expediency rather than equity (Mustafa 2007, Haines 2013, Taylor 2015, Mustafa et al. 2013), thereby entrenching exclusion and reinforcing the political geography of flooding.

Governance weaknesses amplify risk. Policies remain fragmented and poorly implemented, hindered by poor coordination, short-termism, and lack of political commitment (Young 2019, Mustafa 2017). The National Climate Change Policy-2012 (NCCP) and the National Water Policy (NWP), approved in 2018 after a two-decade delay, remain disconnected. The NWP fails to integrate climate concerns or even reference the NCCP (Sattar et al. 2018, Iqbal and Khan 2018). Overlapping mandates among the Ministry of Climate Change (MOCC), Ministry of Water Resources (MOWR), Water and Power Development Authority (WAPDA), Indus River System Authority (IRSA), and Pakistan Meteorological Department (PMD) create inefficiencies, while provincial governments, lacking dedicated climate institutions, rely on under-resourced Environmental Protection Agencies (Young 2019, Mumtaz 2018). These silos result in conflicting decisions over water allocation and monitoring (FODP-WSTF 2012). Groundwater regulation remains a critical gap, with no agency fully accountable (Sattar et al. 2018), and the absence telemetry systems limits real-time flow monitoring (Young 2019). Consequently, responses are reactive and fragmented, with administrative paralysis during emergencies (Young 2019, Cooper 2018). High-profile disputes, such as that over the Kalabagh Dam, underscore the politicization of water management and absence of consensus (Mustafa 2017).

These failures are political as much as technical. Practices like breaching levees or redirecting floodwaters to protect urban centres persist (Mustafa and Wrathall 2011, Inam 2007). Rooted in colonial-era strategies of control (Mustafa et al. 2019), they routinely sacrifice rural regions—home to 64% of the population and over 80% of the poor (MPDSI-Pakistan 2022, Janssen et al. 2023)—to shield economically and politically powerful areas. These areas, often downstream in Sindh and southern Punjab, are both geographically exposed and dependent on agriculture (Rehman et al. 2015). The sector, relying almost entirely on the Indus irrigation system (Chaudhry 2017), employs 37.4% of the workforce (PBS 2021) and contributes 24% to GDP (PBS 2023), face cascading livelihood disruptions (MPDSI-Pakistan 2022). Both in 2010 and 2022, floods devastated crops, livestock, and incomes (MPDSI-Pakistan 2022, Mehmood 2022, Deen 2015), pushing smallholders into informal work and deepening poverty (Ahmad et al. 2020, Ahmad and Ma 2020). Women, landless tenants, and peripheral communities—least represented in decision-making—faced the most severe, prolonged impacts (MPDSI-Pakistan 2022, Syvitski and Brakenridge 2013, Ali et al. 2020). This cycle of exposure and vulnerability, explored further in the section “from exposure to vulnerability: who bears the brunt?”, is shaped by land access, gender, wealth, and political power (Hamidi et al. 2020, Solangi et al. 2022, Hamidi et al. 2020, Schütte and Kreutzmann 2012, Bano et al. 2023).

In sum, Pakistan’s water infrastructure channels both water and vulnerability along historical and political lines. These failures are embedded in decision-making: who decides, for whom, and with what consequences. Persistent reliance on outdated systems and centralized control continues to shape Pakistan’s flood management (Mustafa 2007, Haines 2013, Taylor 2015, Mustafa et al. 2013). Patterns of risk and institutional failure align with critical disaster risk, hydropolitical, and development perspectives (Wisner et al. 2004, Cutter et al. 2003). Addressing Indus Basin flood risk requires more than engineering fixes—it demands rethinking governance, equity, and power to mitigate socially and historically produced risks.

### Institutional fragmentation and systemic governance failures

As Pakistan’s exposure to extreme floods grows, the institutions responsible for managing risk remain fragmented, under-resourced, and reactive (Shah et al. 2019, Majeed 2023, Aslam 2018). While the previous section traced how infrastructure shapes exposure, this section examines how institutional dysfunction amplifies vulnerability. The 2022 floods revealed that extreme weather turns catastrophic not through nature alone, but through institutional failure (Mohmand and Loureiro 2022). This framing is long emphasized by the United Nations (UNDP 2004) and disaster scholarship (Wisner et al. 2004).

Pakistan’s disaster governance problems are longstanding. The 2010 floods, affecting over 24 million people and causing over $10 billion in damages (Rafiq and Blaschke 2012, Shah et al. 2017), revealed similar weaknesses that remain unresolved. Disaster management model is largely reactive, rooted in colonial and post-colonial systems such as the West Pakistan National Calamities Act (1958), which emphasized response over prevention. Disaster response long remained under ad hoc bodies like the Emergency Relief Cell (1971) and similar provincial structures, lacked mandates and capacity for long-term planning. This reactive posture continues to shape Pakistan’s disaster management system, where progress in strengthening federal, provincial, and local capacities has been slow and uneven (NDMA 2012). A formalized system emerged only after repeated catastrophes. The National Disaster Management (NDM) Act (2010) established a hierarchical framework of national, provincial, and district authorities (Ahmed 2013), followed by the National Disaster Management Plan (NDMP) (2012) (NDMA 2012). With a ten-year horizon and PKR 92.02 billion budget, the NDMP aimed to fix early-warning and monitoring failures exposed in 2010 floods (NDMA 2012, Cheema et al. 2016, Ali and Iqbal 2021). Yet implementation stalled amid underfunding, weak coordination, and minimal political ownership. Similarly, the National Flood Protection Plan IV (NFPP-IV, 2017)—a PKR 332 billion initiative—sought to integrate structural and non-structural measures (Federal Flood Commission 2018) but poor collaboration, limited provincial uptake, and narrow infrastructural focus hindered progress (MPDSI-Pakistan 2022, Cheema et al. 2016, Ali and Iqbal 2021). While the NFPP-IV received minor funding, the NDMP remained largely unfunded even a decade later. The National Disaster Risk Management Fund (2017) has also struggled to prioritize vulnerable areas or disburse funds effectively (MPDSI-Pakistan 2022). These persistent gaps delay resilience-building and exacerbate exposure (Harvey et al. 2022).

Part of the problem lies in Pakistan’s fragmented institutional landscape. Responsibilities are scattered across the Federal Flood Commission (FFC), National Disaster Management Authority (NDMA), PMD, and Provincial Disaster Management Authorities (PDMAs)—which operate in silos with overlapping mandates. Local governments, crucial for implementation, remain sidelined by limited autonomy and resources, unable to design or enforce DRR strategies (Khan et al. 2022, Bussell and Fayaz 2017, Manzoor 2022). PDMAs often rely on ad hoc responses (Khan et al. 2022, Shah et al. 2019) and face criticisms after each disaster (Khan et al. 2022, Manzoor 2022, Shah 2023). The result is a governance vacuum. For instance, rural populations often receive little to no warning, undermining one of DRR’s basic functions (Cheema et al. 2016, Ali and Iqbal 2021). Early warning systems and hazard mapping remain fragmented (Shah et al. 2019, Aslam 2018); although some high-risk districts have developed hazard maps (e.g., PDMA 2019), data rarely inform planning due to lack of a centralized data-sharing platform. Similarly, fiscal resilience strategies, though proposed, have seen minimal institutional uptake (MPDSI-Pakistan 2022).

The top-heavy, centralized model limits community-based approaches (Aslam 2018, Jan and Muhammad 2020) despite global recognition of their effectiveness (Maskrey 2011, Shaw and Shaw 2012). While community-based DRR features in national frameworks, it lacks clear implementation pathways, leaving local needs disconnected from policy (Mysorewala 2019). Initiatives like local resilience programs, often donor-driven, are rarely institutionalized, and public participation remains consultative rather than participatory. This weakens trust and prevents policies from addressing ground-level realities (Jan and Muhammad 2020, Rana et al. 2021). Socially vulnerable groups—women, landless farmers, and marginalized communities—remain excluded from decision-making. The echoes of colonial governance are hard to miss: centralized control and elite-focused flood protection at the expense of vulnerable rural populations (Mustafa and Wrathall 2011).

The 2022 floods underscored that Pakistan’s disasters are governance failures as much as climatic ones. Persistent weaknesses—outdated forecasting, poor maintenance, minimal local participation, and lack of fiscal preparedness—have turned recurrent hazards into systemic crises. Overcoming these requires shifting from reactive, centralized models to proactive, inclusive, anticipatory governance: strengthening federal–provincial–local coordination, integrating risk data into planning, investing in early warning and communication systems, empowering local institutions, and supporting iterative, adaptive policy learning (Khan et al. 2022, Kefela 2011, Duyne Barenstein et al. 2012, Mai et al. 2020, Majo 2022). Fiscal mechanisms like micro-insurance, social safety nets, and pre-arranged financing should target the most vulnerable. Ultimately, resilience-building depends not on stronger infrastructure alone but on better governance—coherent, participatory, and equitable. Without institutional reform and political commitment, Pakistan will remain trapped in a recurring cycle of exposure, disaster, and exclusion.

## From exposure to vulnerability: who bears the brunt?

Disasters in Pakistan unfold through deep social, economic, and political inequalities. While earlier sections explored governance failures shaping flood risk, this section examines how vulnerability is socially produced and unevenly distributed across class, gender, and geography. The 2022 floods revealed that smallholders, landless labourers, women, and marginalized groups suffered most—not from hazard exposure alone, but from pre-existing structural disadvantages that limit power, protection, and recovery capacity. In disaster research, this is understood as *social vulnerability*—rooted in poverty, insecure housing, limited credit, healthcare access, and political exclusion (Cutter et al. 2003). In rural Pakistan, floods disproportionately affect low-income households, often living along riverbanks or in informal settlements, with agriculture-dependent communities especially exposed. In 2022, over 2.8 million hectares of cropland were inundated in Sindh alone, destroying harvests (Qamer et al. 2023) and pushing millions into food insecurity (MPDSI-Pakistan 2022, p.23; IPC 2023). Smallholders and tenant farmers, lacking safety nets, resorted to sell assets, migrate, or seek informal work (Ahmad et al. 2020, Ahmad and Ma 2020).

Unequal land ownership remains a central driver of vulnerability. Large landlords and tribal elites control most arable land, while the rural poor farm marginal flood-prone areas, often used as diversion zones during floods (Mustafa and Wrathall 2011, Inam 2007). Such practices protect elite lands while sacrificing poorer communities (Nyborg and Nawab 2017). This inequality is systemic, not incidental. The World Bank (2020) identifies this inequality as a root cause of elite capture and intergenerational poverty (Redaelli 2020). This inequality also distorts recovery: aid and compensation often flow through patronage networks (Government of Pakistan 2022b, Harvey et al. 2022), reinforcing exclusion and dependency (Aijazi 2014).

Poverty both drives and results from vulnerability (Rana et al. 2023, Hamidi et al. 2020, Sinha et al. 2022, Dube et al. 2018). The 2022 floods hit 19 of Pakistan’s 25 poorest districts, pushing over 12 million into poverty (MPDSI-Pakistan 2022, p.19) and escalating food inflation by 45% (UNOCHA 2023). Families facing destitution resorted to harmful coping strategies, including child marriage—45 cases recorded in one Sindh village (Khan Mohammad Mallah) alone (Seigneur 2024). Disasters thus intensify gendered inequalities. Women and girls face structural disadvantages—restricted mobility, limited decision-making power, and unequal access to resources—that hinder evacuation and aid access (Harvey et al. 2022, Mustafa et al. 2019, Prabhu 2022, Ali and Malik 2022). Cultural norms where women eat last (The New Humanitarian 2011), evacuate last, and are often excluded from relief distribution (Sturridge et al. 2022) compound these risks. In 2022 floods, such restrictions often prevented evacuation, as it was considered dishonourable for women to appear publicly or share spaces with unrelated men (Sturridge et al. 2022). Broader inequities reinforce this: women’s labour participation (23%) and literacy (46%) remain far below men’s (81% and 69%) (PBS 2021, Ersado 2023; World Bank 2023), constraining access to information and livelihoods vital for preparedness and recovery (PBS 2021, MPDSI-Pakistan 2022).

Healthcare, sanitation, and water access are also highly unequal (Raza 2023). Before 2022, less than half the population had adequate healthcare; only 33% of women received postnatal care, and child mortality remained high (under-five: 74/1,000; infant: 62; neonatal: 42). Floods damaged 13% of health facilities, leaving 1.2 million more households without access. Over 5.5 million children missed immunizations and 2.8 million lacked maternal services, eroding recent progress toward the Sustainable Development Goals (MPDSI-Pakistan 2022). Sanitation deprivation rose from 17.9% to 21.4%. As women primarily manage household water and waste, they faced heighten exposure to diseases and hygiene-related health risks (MPDSI-Pakistan 2022). Women of childbearing age lacked menstrual clean water, hygiene products, safe disposal options, and gender-segregated spaces in camps (Moazzam 2023; WaterAid 2022) exacerbated their safety and dignity concerns (Tufail et al. 2023). These are not incidental hardships but outcomes of gender-blind disaster planning.

In sum, flood vulnerability in Pakistan is not merely environmental but fundamentally social and political, produced by systemic inequality and exclusion. The 2022 floods reaffirm that disasters emerge not from exposure alone but from the conditions that make certain groups less able to cope and recover. These conditions—e.g., land inequality, gender discrimination, poverty, and neglect of marginalized populations—constitute the very fabric of social vulnerability. Yet, DRR in Pakistan remains focused on physical hazards and asset losses, overlooking deeper structural roots (Mustafa and Wrathall 2011). The 2022 floods highlight the need for a paradigm shift from managing disasters to transforming the systems that create them. DRR strategies must therefore centre social vulnerability: identifying who is most at risk, why, and how institutions can redress inequities. Building resilience requires more than technical or infrastructural fixes; it demands addressing the social production of vulnerability itself. Only by transforming the underlying social structures can Pakistan move from managing disasters to preventing them.

## Systemic risk, governance failures and the future of flood governance

Pakistan’s case shows that flood risk is not purely meteorological but rooted in entrenched social, political, and institutional structures that reproduce vulnerability. Revisiting debates in disaster risk, hydropolitics, and critical development studies, it illustrates how risk is shaped by exclusionary systems, weak governance, and unequal access to protection. As disaster scholarship argues, vulnerability is socially constructed (Wisner et al. 2004, Cutter et al. 2003)—a truth made painfully visible by the 2022 floods. Visible destruction disguises an invisible architecture of risk: a system that protects some while exposing others, shaped by power and marginalization. This systemic production of risk is reflected in Pakistan’s water governance, which still relies on colonial-era infrastructure (Mustafa 2007, Haines 2013, Taylor 2015, Mustafa et al. 2013), centred on command, control, and containment rather than participatory and adaptive approaches (Mustafa 2022). Reform efforts often replicate the same exclusions. For instance, Pakistan’s experience with irrigation management transfer under the 1997 PIDA Act, ended with repeal in Punjab by 2019, offering a cautionary tale. Rather than empowering local actors, many reforms added responsibility without autonomy, failing to uphold principles of collective action and nested governance (Bell 2022). The failure to foster self-organization, transparent rule-making, and effective sanctioning undermined the legitimacy of farmer and water user associations (Wegerich et al. 2014, Chaudhry 2018). Elmore’s (Elmore) concept of “forward mapping” describes these limitations—where policy assumes linear control and predictable results, ignoring fragmented, politicised, and uneven institutional landscape in which outcomes are produced.

Pakistan’s case offers three interlinked lessons: disasters must be understood as politically and socially constructed outcomes; technocratic responses cannot address deep-rooted vulnerability; and resilience must be reimagined as a process rooted in justice, participation, and ecological balance. Yet moving toward such a vision requires more than critique; it demands transformative flood governance (see Fig. 1). As global thinking moves beyond state-centric paradigms (Özerol et al. 2018), Pakistan must shift from hydraulic determinism toward adaptive, community-defined governance models. Recent scholarship calls for focusing on locally relevant ‘problem-sheds’ (Mollinga et al. 2007, Woodhouse and Muller 2017)—a conceptual alternative to watersheds (Fisher 1967, Thomas 2020)—aligning governance with shared challenges rather than imposed administrative or hydraulic boundaries. Problem-sheds identify linked resource issues and communities of common interest, enabling context-specific, cross-sectoral governance. This turn toward flexible, polycentric governance has gained traction globally in both research and practice (Giordano and Shah 2014, Mollinga 2020). Examples from South Africa, Ghana, Thailand, and Brazil demonstrate that multi-actor “issue networks”—around concerns like erosion, livelihoods, or groundwater—serve as effective sites of adaptive governance (Pollard et al. 2023, Boateng and Larbi 2021, Daré et al. 2018, Naivinit et al. 2010, Marques et al. 2020). Such networks emphasize local entry points—water user associations, farmer groups—not as endpoints but as iterative learning spaces (Pahl-Wostl 2009). Integrated Water Resource Management has likewise shifted toward context-specific, hybrid approaches blending formal and informal arrangements (Giordano and Shah 2014). For Pakistan, this means engaging traditional systems like *karez* and *khal panchayats*, which still mediate access and resolve disputes across, especially where formal oversight is weak (Mustafa and Wrathall 2011, Bell 2022). Yet state policies often ignore or override these social institutions, undermining their legitimacy. Similar dynamics have played out e.g. in Bolivia, Nepal, and Ethiopia, where formal frameworks fail to align with customary or indigenous governance systems (Singh et al. 2014, Agramont Akiyama et al. 2022, Mekonnen et al. 2021). Rather than viewing informality as a gap, successful models work to build inclusive, adaptive governance “from below.”

Leadership across levels must evolve. Political leaders must look beyond crisis optics toward long-term reform (FODP-WSTF 2012); institutions must ensure cross-sectoral coordination; and community leadership must promote ownership and accountability (Young 2019, Cooper 2018). Regional cooperation—via the Indus Waters Treaty (India and Pakistan 1960) and the HKH platform—remains vital for joint climate response (Mustafa 2017, Elalem and Pal 2015, Molden et al. 2017), though geopolitical tensions constrain progress (Mustafa 2017). The Water Apportionment Accord (IRSA 1991) should incorporate ecological needs and climate variability (Salik et al. 2016), while Pakistan’s participation in global forums like COP, UNFCCC, GCF, and GEF must be strategically leveraged to secure adaptation finance and influence global climate governance (Gazdar 2005, Mustafa 2010, Salik 2017). Leadership must rest on coherent policy frameworks, often cited as key to governance performance (OECD 2015; CAT 2019). Yet Pakistan’s climate and water policies remain fragmented, with overlapping mandates among FFC, PMD, NDMA, and PDMAs, and limited provincial ownership (Mustafa et al. 2013, Ringler and Anwar 2013, Young 2019, Cheema et al. 2016; UNDP 2017). Such policy proliferation without implementation capacity mirrors challenges across postcolonial states (Siddiqi 2022, Schreiner 2013, Movik 2016). Comparable challenges appear in India’s river-linking plans (Shah et al. 2016), Ethiopia’s dams (Mohammed 2024), and Brazil’s hydropower projects (McCormick et al. 2016), where centralized infrastructures undermine local resilience.

Rethinking effectiveness is crucial. It must be redefined—not by delivery metrics, but by how governance empowers stakeholders, distributes risk, and ensures accountability (McConnell 2010, Bovens and Peters 2002). While the NCCP and NWP provide foundation, a unified, actionable strategy with enforceable accountability is essential (Young 2019, Mumtaz 2018). Policies must be operationalised through participatory frameworks embedding equity, transparency, and legal enforceability (Young 2019, Qureshi et al. 2010, Qamar et al. 2018, Bank 2018, Memon et al. 2020). Understanding systemic flood risk also requires recognising the value of informal institutions, local knowledge, and social learning. Triple-loop learning (Pahl-Wostl 2009) frames governance as a dynamic process where institutions not only pursue goals but rethink assumptions and power relations over time. 

Achieving this transformation requires rethinking knowledge systems. Resilience requires integrating social sciences with engineering and climate science, embracing epistemological pluralism—valuing diverse ways of knowing, participatory governance, local knowledge and lived experiences, and vulnerability mapping, rather than relying solely on technical risk models. Centring justice, equity, and inclusive knowledge in climate risk governance is vital globally, not just in Pakistan. Social vulnerability assessments and community-based DRR better reveal inequalities and enhance equity and responsiveness (e.g., Cutter et al, 2003; Habiba et al., 2013; Fernandez et al., 2012). These tools must be institutionalized to enhance participation, inclusive early warning, and equitable recovery. Adaptive learning spaces can become innovation hubs linking science, policy, and society. Ecological restoration must accompany institutional reform. Wetland recovery, floodplain reconnection, and nature-based buffers are not luxuries but essential for resilience and water security. The Sendai Framework and Paris Agreement both advocate ecosystem-based adaptation, yet to be transformative these must be embedded in governance systems, prioritising justice, inclusivity, and learning. Climate resilience cannot mean returning to the pre-disaster status quo; it must mean moving forward—dismantling marginalisation, decentralising power, and coexisting with water.

To operationalise these principles, Pakistan requires a sequenced and realistic policy pathway. In the short term, improving last‑mile early warning dissemination, repairing critical embankments and drainage networks, improving telemetry systems, and embedding social vulnerability assessments into district‑level planning can substantially reduce immediate exposure. Medium‑term priorities include establishing integrated hydrology–risk data platforms, creating provincial climate–water coordination units, institutionalizing community‑based disaster risk reduction, and strengthening transparency and coordination. Over the long term, structural reforms are essential: transitioning from centralised hydraulic control to polycentric, problem‑shed‑based governance; restoring wetlands, floodplains, and riparian buffers as ecological infrastructure; aligning climate and water policy mandates; and enhancing regional cooperation for shared monitoring and adaptation finance. These interventions directly target the multi‑scalar drivers of systemic risk, linking near‑term vulnerability reduction with long‑term institutional transformation. Successful implementation will depend on strengthening administrative capacity and securing predictable adaptation financing, particularly for provincial and district governments that manage frontline risk. Because governance systems evolve over time, continuous learning, iterative monitoring, and periodic review of progress are essential to ensure that these reforms remain responsive to changing climatic, socio‑economic, and political conditions.

Grounded in Pakistan, these insights have broader relevance across the Global South, where centralized, technocratic systems often suppress local agency and perpetuate risk. Resilience must thus be seen as a governance challenge, not just a technical one. Pakistan’s experience underscores that effective flood governance demands moving from hydraulic control to community-defined “problem-sheds,” recognizing informal institutions as assets, and linking resilience with institutional learning, inclusion, and ecological restoration. These lessons can guide other countries seeking to shift from reactive, infrastructure-heavy responses toward adaptive, inclusive, and just flood governance.

## Conclusion

Disasters are not natural events but the outcomes of political, institutional, and socio-economic systems that determine who is exposed, protected, or left behind. The 2022 floods in Pakistan revealed the consequences of governance failures rooted in colonial infrastructures, deep inequalities, and fragmented policies. Flood risk is socially constructed—produced through historical exclusion, institutional inertia, and technocratic governance. Addressing these systemic drivers requires moving beyond hazard control and reactive relief toward transformative governance that redistributes power and embeds justice, participation, and ecological balance. Resilience cannot rest on the same governance systems that have failed the most marginalized. Flood governance must become a project of transformation—legitimizing informal and traditional institutions, institutionalizing social vulnerability assessments, and embedding community-based disaster risk reduction and adaptive learning as core, not peripheral, strategies. Achieving this transformation demands institutional learning, cross-scale coordination, and genuine inclusion in decision-making spaces where affected communities are empowered rather than merely consulted. Across the Global South, climate change exposes the fragility of centralized, top-down systems. Pakistan’s experience offers broader lessons: flood governance must shift from hydraulic control to community-defined “problem-sheds”; informal institutions should be seen as assets for adaptive capacity; and resilience must be redefined through justice, equity, and transformation—not a return to the status quo. As climate extremes intensify, the cost of inaction will escalate. A just, inclusive, and ecologically grounded model of flood governance is essential for Pakistan—and for all societies navigating the climate crisis.

## Data Availability

No datasets were generated or analysed during the current study.
